# Leveraging Mathematical Modeling to Quantify Pharmacokinetic and Pharmacodynamic Pathways: Equivalent Dose Metric

**DOI:** 10.3389/fphys.2019.00616

**Published:** 2019-05-22

**Authors:** Matthew T. McKenna, Jared A. Weis, Vito Quaranta, Thomas E. Yankeelov

**Affiliations:** ^1^Vanderbilt University Institute of Imaging Science, Vanderbilt University, Nashville, TN, United States; ^2^Department of Biomedical Engineering, Vanderbilt University, Nashville, TN, United States; ^3^Department of Biomedical Engineering, Wake Forest School of Medicine, Winston-Salem, NC, United States; ^4^Comprehensive Cancer Center, Wake Forest Baptist Medical Center, Winston-Salem, NC, United States; ^5^Department of Cancer Biology, Vanderbilt University School of Medicine, Vanderbilt University, Nashville, TN, United States; ^6^Department of Biomedical Engineering, The University of Texas at Austin, Austin, TX, United States; ^7^Department of Diagnostic Medicine, Dell Medical School, The University of Texas at Austin, Austin, TX, United States; ^8^Department of Oncology, Dell Medical School, The University of Texas at Austin, Austin, TX, United States; ^9^Oden Institute for Computational and Engineering Sciences, The University of Texas at Austin, Austin, TX, United States; ^10^Livestrong Cancer Institutes, The University of Texas at Austin, Austin, TX, United States

**Keywords:** mathematical modeling, breast cancer, pharmacokinetic modeling, pharmacodynamics, doxorubicin, treatment response

## Abstract

Treatment response assays are often summarized by sigmoidal functions comparing cell survival at a single timepoint to applied drug concentration. This approach has a limited biophysical basis, thereby reducing the biological insight gained from such analysis. In particular, drug pharmacokinetic and pharmacodynamic (PK/PD) properties are overlooked in developing treatment response assays, and the accompanying summary statistics conflate these processes. Here, we utilize mathematical modeling to decouple and quantify PK/PD pathways. We experimentally modulate specific pathways with small molecule inhibitors and filter the results with mechanistic mathematical models to obtain quantitative measures of those pathways. Specifically, we investigate the response of cells to time-varying doxorubicin treatments, modulating doxorubicin pharmacology with small molecules that inhibit doxorubicin efflux from cells and DNA repair pathways. We highlight the practical utility of this approach through proposal of the “equivalent dose metric.” This metric, derived from a mechanistic PK/PD model, provides a biophysically-based measure of drug effect. We define equivalent dose as the functional concentration of drug that is bound to the nucleus following therapy. This metric can be used to quantify drivers of treatment response and potentially guide dosing of combination therapies. We leverage the equivalent dose metric to quantify the specific intracellular effects of these small molecule inhibitors using population-scale measurements, and to compare treatment response in cell lines differing in expression of drug efflux pumps. More generally, this approach can be leveraged to quantify the effects of various pharmaceutical and biologic perturbations on treatment response.

## Introduction

The parameterization of *in vitro* treatment response data is central to biomarker and drug discovery and the quantitative study of cancer therapies. With recent exceptions (Hafner et al., 2016; Harris et al., 2016), investigation of treatment response *in vitro* has been limited to cell survival assays that assess cell viability at a single, specified timepoint following treatment with a temporally constant concentration of drug. A range of drug concentrations are evaluated in these assays, and the results are conventionally summarized by Hill function parameters, which quantify cell survival with respect to applied drug concentration (Fallahi-Sichani et al., 2013). While this approach has yielded significant insights into cancer biology, it is fundamentally limited by the coarseness of parameters used to summarize treatment response. In particular, these parameters do not explicitly characterize the dynamics of treatment and subsequent response. Further, response metrics are reported relative to the extracellular concentration of drug in the assay, overlooking drug exposure times and variable cell line pharmacologic properties. This not only impairs analysis of *in vitro* treatment response data, but also presents a challenge in translating these therapies *in vivo*.

There are a host of biochemical processes that modulate a tumor cell's response to therapy. For example, the accumulation of drug within cells can be altered by drug metabolism or modification of surface proteins that regulate drug flux through the membrane (Larsen and Skladanowski, 1998; Larsen et al., 2000). Indeed, the multi-drug resistance protein 1 (MDR1) is a well-studied mechanism of resistance to cytotoxic therapies (Clarke et al., 2005). This ATP-dependent pump actively effluxes drug from cells, decreases drug accumulation within cells, and confers resistance to anthracyclines, taxanes, and several other agents (Mechetner et al., 1998). Similarly, pharmacodynamic response to therapies can be altered through modulation of signaling pathways downstream of the therapeutic target. With respect to DNA-damaging agents, changes in DNA repair pathways, which are activated in response to treatment, can alter sensitivity to those agents (Fink et al., 1998; Bouwman and Jonkers, 2012). For example, DNA-dependent protein kinase (DNA-PK) plays a major role in the repair of double strand DNA breaks *via* non-homologous end joining (Smith and Jackson, 1999). Increased expression of DNA-PK has been shown to confer resistance to doxorubicin, an anthracycline commonly used clinically (Shen et al., 1998). Fundamentally, cell line-specific pharmacokinetic and pharmacodynamic properties, such as those described above, drive observed treatment responses. Using conventional methods, these processes are conflated by the parameters used to summarize *in vitro* dose response data (Prentice, 1976; Fallahi-Sichani et al., 2013). The resulting parameters are imprecise measures of drug efficacy, which limits the biological insights to be gained from the data.

More precise technologies are required to advance systems approaches to studying cellular response to therapy (Anderson and Quaranta, 2008). We posit that a mechanistic, mathematical modeling framework is essential to maximize the knowledge gained through treatment response studies (Yankeelov et al., 2013, 2015). In this paradigm, biologically-motived mathematical models are constructed to describe observed behaviors of the system under investigation. The model is then fit to experimental data, yielding a set of parameter values that provide mechanistic insight into observed data. There exist several models in the literature that explicitly incorporate drug pharmacokinetics (PK) and pharmacodynamics (PD) to describe treatment response. *In vitro*, transit compartment models have been used to describe the temporal relationship between drug application and effects (Lobo and Balthasar, 2002). More biologically-motivated PK/PD models have been employed to study specific pharmacokinetic and pharmacodynamic parameters (Lankelma et al., 2003, 2013). PK/PD models have also been developed to investigate treatment response *in vivo* (Simeoni et al., 2004; Sanga et al., 2006; Wang et al., 2015). Recently, we proposed and validated a coupled PK/PD model of doxorubicin treatment response *in vitro* (McKenna et al., 2017). The model incorporates measured doxorubicin pharmacokinetics and pharmacodynamics and predicts response to a specified treatment timecourse on a cell line-specific basis. The model behaves consistently across a wide spectrum of treatment protocols and cell lines, thereby demonstrating that the response dynamics of cancer cell lines to doxorubicin is predictable within this framework. Specifically, the PK model-estimated concentration of doxorubicin bound to the cell nucleus is predictive of cell line pharmacodynamic rates. We further noted a mismatch of drug uptake and response among the investigated cell lines, suggesting that each cell line has an intrinsic sensitivity to stress induced by doxorubicin. By explicitly modeling both drug uptake and subsequent effect, these processes can be independently quantified to study each component of treatment response (McKenna et al., 2017).

It is the goal of the present effort to demonstrate the utility of a mechanistic, mathematical modeling framework in quantifying treatment response and PK/PD pathways. We leverage mathematical models to filter experimental data to yield quantitative measures of specific cellular processes. Specifically, we experimentally perturb doxorubicin pharmacokinetics and pharmacodynamics with chemical inhibitors of each process. We modulate pharmacokinetics in an MDR1 over-expressing cell line and modulate pharmacodynamics *via* DNA-PK in a BRCA1-mutated cell line. These data are analyzed with the proposed PK/PD model to yield quantitative measures of these pathways. We further illustrate the utility of our approach by proposing the equivalent dose metric, which we derived from the PK model. The equivalent dose is analogous to that in radiation therapy, which is used to compare radiation fractionation schedules (Fowler, 1992). In the context of chemotherapy, we define equivalent dose as a functional measure of drug exposure. We specify that for a given equivalent dose, treatment response dynamics are similar. As this approach accounts for variable pharmacologic properties, we posit that it allows for more precise comparisons among cell lines relative to metrics based on extracellular drug concentration. We demonstrate this experimentally through comparison of treatment response in cell lines differing only in MDR1 expression. The modeling-based framework proposed in this work can be leveraged to more precisely quantify the effects of various pharmaceutical and biologic perturbations on treatment response.

## Materials and Methods

### Mathematical Model of Doxorubicin Treatment Response

Doxorubicin is an anthracycline that remains standard-of-care therapy for several cancers (Tacar et al., 2013). Ultimately, doxorubicin induces a host of cellular stress responses which either inhibit further DNA synthesis allowing for cellular recovery, or initiate a cascade leading to cell death (Gewirtz, 1999). At high doxorubicin concentrations, extensive DNA damage often results in cell death *via* apoptosis. Low to moderate concentrations of doxorubicin induce cell senescence and cell death *via* mitotic catastrophe (Chang et al., 1999; Eom et al., 2005). Whereas, apoptosis is immediate (on the order of hours to days), mitotic catastrophe is a relatively protracted process (on the order of several days).

We previously developed and validated a parsimonious treatment response model to describe doxorubicin pharmacokinetics and pharmacodynamics (McKenna et al., 2017). Briefly, a three-compartment model was employed to describe the uptake and binding of doxorubicin in cancer cells. This process is modeled *via* mass conservation through Equations (1–3):

(1) $$\begin{array}{r} \frac{dC_{E}\left(t\right)}{dt}=k_{FE}\frac{v_{I}}{v_{E}}C_{F}\left(t\right)-k_{EF}C_{E}\left(t\right) \end{array}$$

(2) $$\begin{array}{r} \frac{dC_{F}\left(t\right)}{dt}=k_{EF}\frac{v_{E}}{v_{I}}C_{E}\left(t\right)-k_{FE}C_{F}\left(t\right)-k_{FB}C_{F}\left(t\right) \end{array}$$

(3) $$\begin{array}{r} \frac{dC_{B}\left(t\right)}{dt}=k_{FB}C_{F}\left(t\right) \end{array}$$

where *C*_*E*_ (*t*), *C*_*F*_ (*t*), and *C*_*B*_ (*t*) are the concentrations of doxorubicin in the extracellular, free, and bound compartments, respectively, at time *t*. The free compartment represents drug that has diffused into the cell, while the bound compartment represents drug that has bound to DNA. The *k*_*ij*_ parameters are rate constants that describe the movement of doxorubicin between the *i*^*th*^ and *j*^*th*^ compartments; for example, *k*_*FE*_ describes the rate of drug transfer from the free, intracellular compartment to the extracellular compartment. Similar definitions apply to *k*_*EF*_ and *k*_*FB*_. The volumes of the extracellular and intracellular compartments are denoted by *v*_*I*_ and *v*_*E*_, respectively (see Table 1 for a full list of model parameter definitions). We note that in this model, several intracellular processes, including doxorubicin metabolism and dissociation from DNA, are not explicitly considered. In previous work (McKenna et al., 2017), we evaluated the performance of several candidate models in describing our experimental data (described in section Doxorubicin Uptake Imaging and Image Processing) with the Akaike information criterion. Of the proposed models, we found that Equations (1–3) best balanced accuracy and the number of model parameters.

**Table 1:** Model parameter definitions.

**Model Parameter**	**Units**	**Definition**
*k_*EF*_*	h^−1^	Rate of drug influx into cell
*k_*FE*_*	h^−1^	Rate of drug efflux from cell
*k_*FB*_*	h^−1^	Mixed rate of drug binding and DNA repair
*C_*E*_*	nM	Extracellular doxorubicin concentration
*C_*I*_*	nM	Intracellular, extranuclear doxorubicin concentration
*C_*B*_*	nM	Concentration of doxorubicin bound to the nucleus
*D_*eq*_*	nM	Equivalent dose
*N_*TC*_*	Count	Number of cells
*k_*p*_*	h^−1^	Proliferation rate of cells
θ	Count	Carrying capacity of experimental system
*k_*d, a*_*	h^−1^	Death rate assumed in Equation (5)
*k_*d, b*_*	h^−1^	Death rate assumed in Equation (6)
*r*	h^−1^	Rate of induction and decay of death rate in Equation (6)

*Complete listing of model parameter definitions*.

A logistic growth model, Equation (4), modified by either of two empirical time-dependent response functions, Equations (5, 6), reflecting distinct mechanisms of cell death, was proposed to describe population level response to doxorubicin therapy as follows:

(4) $$\begin{array}{r} \frac{dN_{TC}\left(t\right)}{dt}=\left(k_{p}-k_{d}\left(t,D\right)\right)N_{TC}\left(t\right)\left(1-\frac{N_{TC}\left(t\right)}{\theta \left(D\right)}\right) \end{array}$$

(5) $$k_{d}\left(t,D\right)=\{\begin{array}{cc} 0 & t<0\\ k_{d,a}\left(D\right) & t\geq 0 \end{array}$$

(6) $$k_{d}\left(t,D\right)=\{\begin{array}{cc} 0 & t<0\\ k_{d,b}\left(D\right)r\left(D\right)te^{1-r\left(D\right)t} & t\geq 0 \end{array}$$

where *k*_*p*_ and *k*_*d*_ are the proliferation and dose-specific death rates, respectively, *D* is the delivered dose [defined to be the bound concentration of drug, *C*_*B*_, calculated with Equations (1–3)], *r* is a dose-specific constant describing the rate at which treatment induces an effect, θ is the dose-specific carrying capacity describing the maximum number of cells that can be observed in the experimental system, and *N*_*TC*_(*t*) is the number of cells at time *t*. Logistic growth models have traditionally been used to describe growth of a variety of biological species whose total size is limited (Gerlee, 2013; Jarrett et al., 2018). This equation accurately describes our experimental system (described in section Doxorubicin Treatment Response Imaging), in which cell population is limited by the surface area of the experimental platform. Prior to treatment (i.e., *t* < 0), cells are modeled to have a constant proliferation rate, *k*_*p*_. Following treatment at *t* = 0, Equation (5) assumes an immediate induction of a stable, post-treatment death rate (*k*_*d, a*_). Equation (6) allows for a smooth induction of drug effect following treatment to a maximum death rate of *k*_*d, b*_, while ultimately allowing for recovery of the cell population. The dynamics of this induction and decay is governed by *r*. A weighted averaging approach is used to incorporate both Equations (5, 6) in the treatment response model. This model was designed to describe cell death *via* apoptosis Equation (5) and mitotic catastrophe Equation (6) and fit experimental data well. Further details on the model can be found in McKenna et al. (2017).

### Equivalent Dose

**Figure 1:**
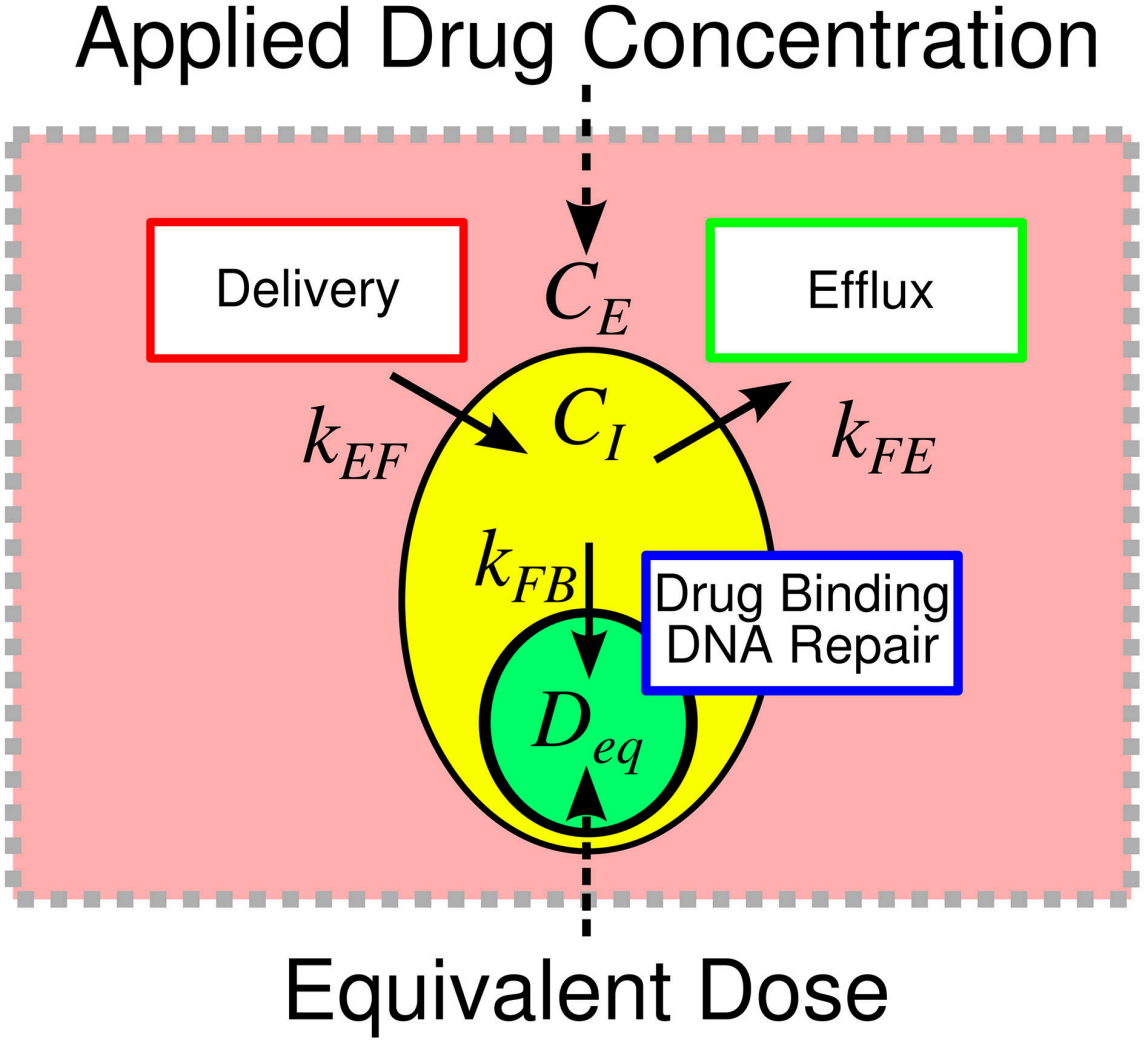
Overview of equivalent dose metric. The response to therapy is determined by the applied drug concentration and cell-specific pharmacologic properties. Traditionally, therapeutic response is summarized relative to the applied extracellular concentration of drug. We propose the equivalent dose metric, *D*_*eq*_, to summarize the contributions of various pharmacologic properties in shaping treatment response. We define equivalent dose as a measure of the functional drug concentration that enters the cell. The equivalent dose is calculated through a mechanistic biophysical model that considers several sources of variability in shaping treatment response. The metric consolidates variable drug uptake (quantified with *k*_*EF*_), efflux (quantified with *k*_*FE*_), and binding (quantified with *k*_*FB*_) into a single descriptor of treatment. The equivalent dose summarizes pharmacologic properties to provide biological insight into treatment response and allows for more precise comparison of treatment response among cell lines.

We define equivalent dose (*D*_*eq*_) as a functional measure of therapy, a summary statistic connecting the amount of drug delivered with the biological effect of that drug. In the context of doxorubicin therapy, we define equivalent dose as the functional concentration of drug that is bound to the nucleus following therapy. To calculate *D*_*eq*_ for a specified treatment condition (i.e., extracellular drug concentration timecourse), Equations (1–3) are populated by cell-line- and treatment-specific *k*_*EF*_, *k*_*FE*_, and *k*_*FB*_ parameters derived from experimental data (described below). The model is then simulated using the experimentally-defined treatment condition. *D*_*eq*_ is the maximum concentration of bound drug (*C*_*B*_) as predicted by the simulation. We hypothesize that the equivalent dose metric can account for variable cell line pharmacologic properties through its explicit consideration of *k*_*EF*_, *k*_*FE*_, and *k*_*FB*_ rates, and it can be leveraged to quantify the effect of agents that modulate those properties. Notably, in proposing the equivalent dose, we lump pharmacodynamic effects into the *k*_*FB*_ term. Specifically, *k*_*FB*_ is a mixed measure of doxorubicin binding and DNA repair and describes the functional net binding rate. The equivalent dose is illustrated in Figure 1.

### Cell Lines

The MDA-MB-468 and SUM-149PT cell lines were obtained through American Type Culture Collection (ATCC, http://www.atcc.org) and maintained in culture according to ATCC recommendations. Cell lines were passaged no more than 30 times before being discarded. To facilitate automated image analysis for identifying and quantifying individual nuclei in time-lapsed microscopy experiments (described below), each cell line was modified to express a histone H2B conjugated to monomeric red fluorescent protein (H2BmRFP; Addgene Plasmid 18982) as previously described (Quaranta et al., 2009; Tyson et al., 2012).

To specifically modulate doxorubicin pharmacokinetics, the H2BmRFP-expressing MDA-MB-468 cell line (MDA-MB-468_H2B_) was transduced to express a green fluorescent protein (GFP)-tagged MDR1 protein (ABCB1 gene, Origene Technologies, Rockville, MD). Following transduction, the cell line was cultured in 100 nM doxorubicin for 2 weeks to select a doxorubicin-resistant phenotype (MDA-MB-468_MDR1_). These cells were serially imaged to ensure that all surviving cells stably expressed GFP.

The SUM-149PT cell line possesses a BRCA1 2288delT mutation (Elstrodt et al., 2006). BRCA1 is involved in maintaining genome stability through its role in repairing double strand DNA-breaks *via* homologous recombination (Gudmundsdottir and Ashworth, 2006). The BRCA1 mutation causes an increased reliance on alternate DNA damage repair pathways, such as non-homologous end joining (Farmer et al., 2005). The DNA damage repair pathway mediated by DNA-PK was targeted with a small molecule inhibitor to specifically modulate doxorubicin pharmacodynamics in the SUM-149PT cell line.

### Chemicals

Doxorubicin was purchased from Sigma Aldrich (St. Louis, MO) and dissolved to a 1 mM stock concentration in sterile saline for subsequent experiments. Tariquidar (TQR) is a third-generation MDR1 inhibitor that non-competitively inhibits MDR1 function (Mistry et al., 2001). TQR is leveraged to modulate doxorubicin pharmacokinetics in the MDA-MB-468_MDR1_ cell line. NU7441 is a DNA-PK inhibitor that has been investigated as a means to improve treatment response to DNA-damaging agents (Zhao et al., 2006; Ciszewski et al., 2014). NU7441 is used to modulate doxorubicin pharmacodynamics in the SUM-149PT cell line. TQR and NU7441 were both purchased from Selleckchem (Boston, MA). Each was dissolved to a 1 mM stock concentration in DMSO. We subsequently refer to these therapies (TQR and NU7441) as sensitizers. All solutions were stored in 250 μL aliquots at -80°C.

### Doxorubicin Uptake Imaging and Image Processing

Time resolved fluorescent microscopy was employed to characterize the uptake of doxorubicin by each cell line (MDA-MB-468_H2B_, MDA-MB-468_MDR1_, and SUM-149PT) using a modification of the previously-published drug uptake assay (McKenna et al., 2017). The method leverages the intrinsic fluorescence of doxorubicin to quantify the movement of doxorubicin from the extracellular space into cells. Briefly, each cell line was introduced into 96-well microtiter plates at ~10,000 cells per well. Each well was imaged at 20–25 min intervals *via* fluorescent microscopy with a 20 × objective in 2 × 2 image montages on a BD Pathway 855 Bioimager (BD Biosciences, San Jose, CA). Imaging began 1 h prior to and continued for approximately 24 h following application of 1 μM of doxorubicin. After 8 h, doxorubicin was removed *via* media replacement. This timeframe allowed for an extended observation of drug uptake without inducing morphological changes and cell death that would limit the effect of the measurement. To measure the effect of TQR and NU7441 on drug uptake kinetics in the MDA-MB-468_MDR1_ and the SUM-149PT cell lines, respectively, each sensitizer was applied over a range of concentrations (250–2 nM for TQR and 2 μM−16 nM for NU7441 both *via* a 2-fold dilution series) 1 h prior to doxorubicin application. At least three replicates of each treatment condition were collected.

The collected images were subsequently post-processed to correct for uneven background illumination and to isolate the contribution of each fluorophore in the experiment. First, the illumination function for each image was estimated (Jones et al., 2006). The image is defined:

$$\begin{array}{l} I=L\left(C+b\right) \end{array}$$

where *I* is the image, *L* is the illumination function, *C* is signal from cells, and *b* is the background. The signal from cells was removed from each image through use of a median disc filter with a radius of 50, isolating *b*. To estimate *L*, the background-only images in each well were averaged over all timepoints. A smooth surface was fit to this averaged image, and the surface was normalized to a maximum value of 1. Each image in the time series was divided by this surface (*L*) to correct for uneven illumination. Following illumination correction, a threshold-based approach was used to segment each cell.

To account for the various fluorophores in the experiment (H2BRFP, MDR1GFP, and doxorubicin), a linear unmixing approach was employed to isolate the signal from each fluorophore to more precisely quantify doxorubicin accumulation (Zimmermann, 2005). The approach leverages spectral imaging data collected at multiple excitation and emission wavelengths to isolate the signal from each fluorophore. This method can also be used for background subtraction by modeling the background (here, the signal from cell culture media) as an additional fluorophore. For these experiments, we define four fluorophores of interest: MDR1GFP, doxorubicin, H2BRFP, and background. The observed images are modeled as a linear combination of the signals from each of these fluorophores:

$$\begin{array}{l} \left[\begin{array}{cccc} S_{H2B} & S_{MDR} & S_{Dox} & S_{background} \end{array}\right]\;T_{4\times n}=\left[\begin{array}{cccc} I_{1} & I_{2} & \ldots & I_{n} \end{array}\right] \end{array}$$

where *S*_*H*2*B*_ is the signal from the H2BRFP, *S*_*MDR*_ is the signal from the GFP-tagged MDR1, *S*_*Dox*_ is the signal from doxorubicin, and *S*_*background*_ is the background signal from cell culture media. *T* is the transformation matrix that estimates the contribution from each fluorophore in creating each image *I*. In this work, five images (*n* = 5) were collected at each timepoint. The excitation, dichroic, and emission filters for each image are listed in Table 2.

**Table 2:** Filter settings.

**Image**	**Excitation (nm)**	**Dichroic (nm)**	**Emission (nm)**
*I_1_*	470/40	515, longpass	515, longpass
*I_2_*	470/40	515, longpass	570, longpass
*I_3_*	470/40	515, longpass	575/25
*I_4_*	470/40	515, longpass	540/50
*I_5_*	548/20	595, longpass	645/75

*Fluorescence imaging filter sets used to collect pharmacokinetics data*.

To construct *T*, images of each fluorophore were collected from control samples. Specifically, control images of GFP, H2BRFP-positive cells, doxorubicin, and background were collected. For each fluorophore, the image with the highest intensity is assumed to be the true image; i.e., the corresponding entry in *T* is set to 1. The relative intensity of the other four images with respect to the true image are then estimated. This normalized spectrum is deposited into the row of *T* corresponding to the current fluorophore. *T* is estimated at each timepoint to compensate for any temporal changes in fluorophore intensity.

With an estimate of *T* and a spectral image set for each well at each timepoint, the underlying signals (i.e., *S*_*H*2*B*_, *S*_*MDR*_, *S*_*Dox*_, *S*_*background*_) can be estimated using QR decomposition [implemented in MATLAB (Mathworks, Natick, MA)]. This can be done on a per-pixel basis as shown in Supplementary Figure 1. However, as we are only interested in the intracellular and extracellular doxorubicin signals, the average value from each image in the intracellular and extracellular (*I*_*i, I*_*, I*_*i, E*_) space was calculated using a cell segmentation (as detailed above). Each signal can then be recovered:

$$\begin{array}{l} \left[\begin{array}{cccc} S_{H2B,I} & S_{MDR,I} & S_{Dox,I} & S_{background,I}\\ S_{H2B,E} & S_{MDR,E} & S_{Dox,E} & S_{background,E} \end{array}\right]\;T_{4\times n}=\left[\begin{array}{ccc} I_{1,I} & \ldots & I_{5,I}\\ I_{1,E} & \cdots & I_{5,E} \end{array}\right] \end{array}$$

where *S*_*Dox, I*_ and *S*_*Dox, E*_ are the signals from doxorubicin in the intracellular and extracellular spaces, respectively. Similar definitions apply for the other signals *S*.

Finally, *S*_*Dox*_ is converted into doxorubicin concentration. We assume that doxorubicin signal is linearly proportional to its concentration, [*Dox*] (McKenna et al., 2017):

$$\begin{array}{l} S_{Dox}=a\left[Dox\right]+b \end{array}$$

To calibrate this model, images are collected on a series of wells containing a range of known doxorubicin concentrations. Estimates of *a* and *b* were obtained by fitting the doxorubicin signal equation to these control data. The image processing pipeline is illustrated in Supplementary Figure 1.

### Doxorubicin Treatment Response Imaging

Using the previously-published dose-response assay, each cell line was treated with a range of doxorubicin concentrations (5,000–10 nM *via* a 2-fold dilution series) for 24 h as monotherapy. Additionally, the sensitizing effects of TQR and NU7441 in the MDA-MB-468_MDR1_ and the SUM-149PT cell lines, respectively, were investigated by applying those therapies over a range of concentrations 1 h prior to application of doxorubicin. TQR concentrations in a 2-fold dilution series from 250 to 2 nM were used for the MDA-MB-468_MDR1_ cell line, and NU7441 concentrations in a 2-fold dilution series from 2 μM to 15 nM were used for the SUM-149PT cell line. These combination studies were each performed at three doxorubicin concentrations. All drug (doxorubicin and sensitizer) was removed from each well *via* media replacement at 24 h. These cells were imaged daily *via* fluorescent microscopy for at least 15 days following treatment. For these studies, fluorescence microscopy images were collected using a Synentec Cellavista High End platform (SynenTec Bio Services, Münster, Germany) with a 20 × objective and tiling of 25 images. To generate images, the H2BmRFP fluorophores were excited with 529 nm light for 650 ms, and emissions were collected at 585 nm. Nuclei were segmented and counted in ImageJ (http://imagej.nih.gov/ij/) using a previously-described, threshold-based method (Frick et al., 2015) to quantify cell population. Six replicates of each treatment condition were collected. Media was refreshed every 3 days for the duration of each experiment to ensure sufficient growth conditions for surviving cells. Data were manually truncated when cell populations reached carrying capacity. At this point, signals from neighboring nuclei overlap, and the cell counting algorithm becomes unreliable.

### Model Fits

The three-compartment model described in Equations (1–3) was fit to the uptake data under each treatment condition (doxorubicin monotherapy and doxorubicin combination with sensitizer) for each cell line using a non-linear least squares optimization implemented in MATLAB. Of note, each cell line is assumed to have a single set of compartment model parameters (*k*_*EF*_, *k*_*FE*_, and *k*_*FB*_) for each sensitizer concentration; i.e., a parameter set for doxorubicin monotherapy and a set for each sensitizer concentration. The mean errors of the best-fit model across all timepoints and treatment conditions with respective standard deviations are reported. Similarly, the pharmacodynamic model described by Equations (4–6) was fit to the dose response data from all treatment conditions (i.e., doxorubicin monotherapy and doxorubicin combination with sensitizer) for each cell line. Each treatment condition in each cell line was fit independently, yielding cell line- and treatment condition-specific parameter values. This was also accomplished through a non-linear least squares optimization implemented in MATLAB, and we report the mean percent errors of the best-fit models across all timepoints. For additional details on the model fitting procedure see McKenna et al. (McKenna et al., 2017).

### Measurement of Pharmacologic Properties With Equivalent Dose

We assume, by definition, that each unique treatment response timecourse corresponds to a specific equivalent dose. As the equivalent dose is perfectly known for doxorubicin monotherapy [i.e., the equivalent dose is simply *C*_*B*_, which can be directly calculated with *k*_*FE*_, *k*_*EF*_, and *k*_*FB*_ values measured from drug uptake studies], the equivalent dose for co-treatment conditions can be estimated by comparing treatment response dynamics from co-treatment conditions to those from doxorubicin monotherapy treatments. With appropriate experimental design to isolate each equivalent dose parameter (i.e., *k*_*FE*_, *k*_*EF*_, and *k*_*FB*_), this approach can quantify the effect of each sensitizing agent on their PK/PD pathway. Specifically, by assuming the effect of each sensitizing therapy is limited to a single equivalent dose parameter, the effect of TQR on *k*_*EF*_ and the effect of NU7441 on *k*_*FB*_ can be measured. As response under all treatment conditions (i.e., doxorubicin monotherapy and co-treatment with a sensitizer) can by summarized by the parameters in Equations (4-6) (i.e., *p* = [*k*_*d, a*_, *k*_*d, b*_, *r*]), we use model parameters to compare treatment response timecourses.

The response parameters (*p*) from doxorubicin monotherapy experiments are first interpolated with respect to equivalent dose *via* a local linear approach. This yields a continuous set of parameters (*p*_*est*_) across all possible equivalent doses in the range from no treatment to maximal doxorubicin dose. The fit parameter values (*p*_*fit*_) for each of the *m* co-treatment conditions are then matched to the interpolated parameters from doxorubicin-only treatment conditions (*p*_*est*_) to estimate the equivalent dose (*D*_*est*_) for each co-treatment condition. Specifically, *D*_*est*_ is the set of equivalent doses that correspond to the best matches between *p*_*fit*_ and *p*_*est*_ in the *L*_2_ norm sense (i.e., min ||*p*_*est*_ − *p*_*fit*_||_2_). This process is illustrated in Supplementary Figure 2. The following constrained objective function, *G* (*k*_*x*_), can then be used to estimate *k*_*x*_ (the equivalent dose parameter under investigation; e.g., *k*_*EF*_ and *k*_*FB*_) for each of the *n* sensitizer concentrations:

(7) $$\begin{array}{l} G\left(k_{x}\right)=\underset{k_{x}}{\min}\overset{m}{\underset{i=1}{\sum }}{\left(D_{est,i}-D_{i}\left(k_{x}\right)\right)}^{2}\\ such\;that{\;k}_{x,q+1}-k_{x,q}\geq 0\;\forall q=\left[1,\ldots ,n\right] \end{array}$$

where the *D*_*i*_ is the equivalent dose calculated for each co-treatment condition as described below, and *D*_*est, i*_ is the estimated equivalent dose for the *i*^*th*^ co-treatment condition. Specifically, in calculating *D*_*i*_for the NU7441 experiments, we fix *k*_*EF*_ and *k*_*FE*_ values and optimize *k*_*FB*_ values corresponding to each sensitizer concentration in the co-treatment conditions. The constraints in the objective function ensure that *k*_*FB*_ increases monotonically with sensitizer concentration. Similarly, for the TQR experiments, we fix *k*_*EF*_ and *k*_*FB*_ and optimize *k*_*FE*_ for each sensitizer concentration. This objective function was minimized *via* a constrained optimization routine implemented in MATLAB. The non-parametric interpolation and optimization procedures were utilized as we did not assume any functional relationships between model parameters and equivalent dose. While this fitting procedure could have been made more robust by proposing such functional relationships, we implemented this non-parametric approach to allow for greater generalizability.

### Comparison of Cell Lines With Equivalent Dose

As the MDA-MB-468_MDR1_ line was engineered from the MDA-MB-468_H2B_ line, we hypothesize that the response of these cell lines to doxorubicin therapy is not significantly different when compared *via* equivalent dose. Specifically, the mechanism of action of MDR1 is to increase drug efflux, which effectively reduces the equivalent dose in the MDA-MB-468_MDR1_ line for a given treatment timecourse. Indeed, the proposed equivalent dose metric was developed to account for the differing pharmacokinetic properties between these cell lines to more precisely compare their respective responses to therapy. To test this hypothesis, survival of the parental MDA-MB-468_H2B_ cell line is compared to that of the MDA-MB-468_MDR1_ cell line. This comparison is made utilizing a conventional treatment response assay in which survival is assessed 72 h following treatment. Specifically, each cell line was treated with a range of doxorubicin concentrations (5,000–10 nM *via* a 2-fold dilution series) for 24 h as monotherapy, and survival was assessed *via* cell counting. Survival data for each cell line was fit with a pair of Hill functions. The first of these Hill functions assumed the dose to be the applied doxorubicin concentration. The second utilized the equivalent dose (*D*_*eq*_) calculated with cell-line specific *k*_*EF*_, *k*_*FE*_, and *k*_*FB*_ values. We report the *EC*_50_ (drug concentration at half-maximal effect) for each cell line as measured *via* extracellular doxorubicin concentration and equivalent dose.

## Results

### Treatment Response in MDA-MB-468_MDR1_ Cell Line

**Figure 2:**
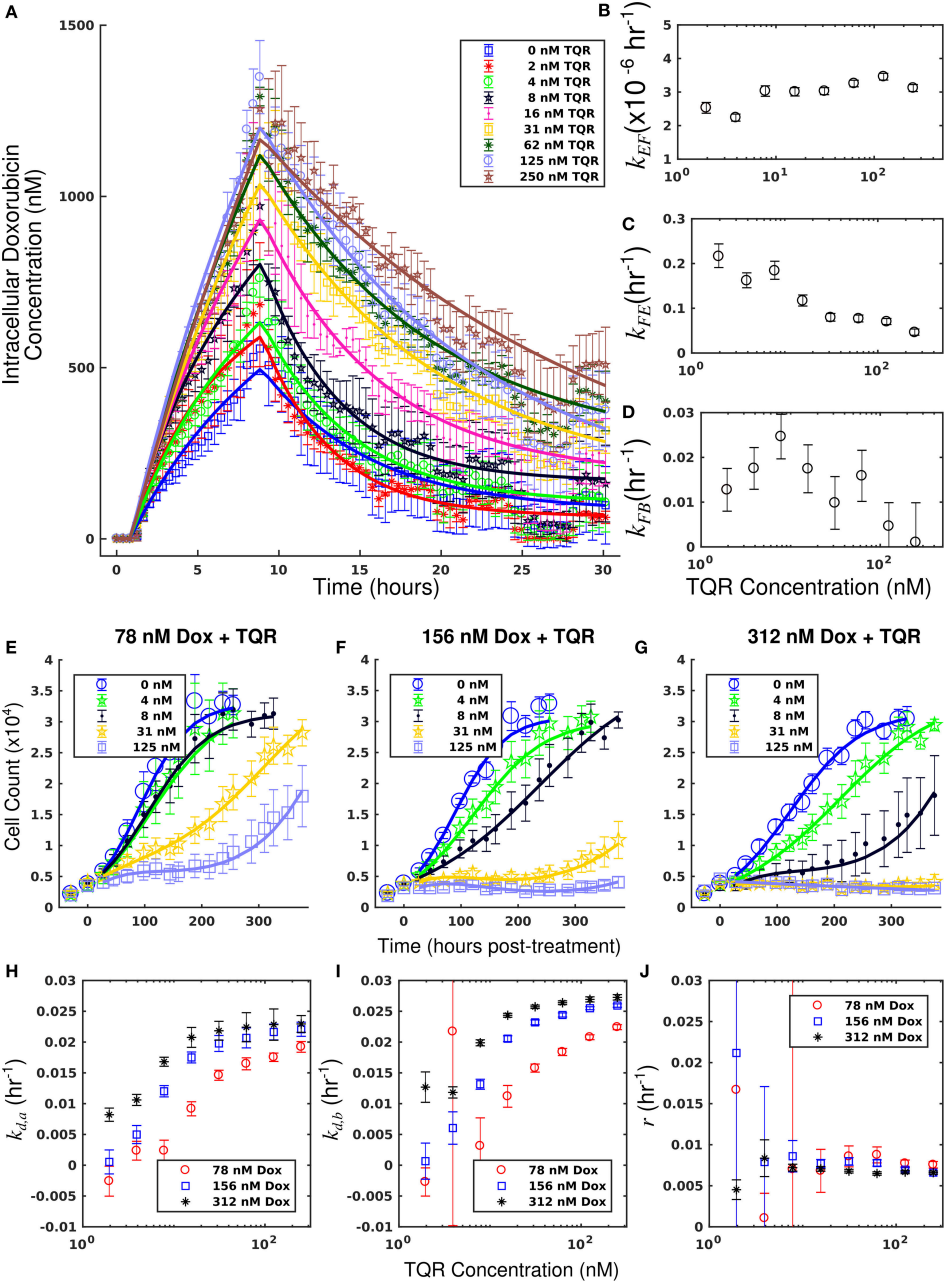
Doxorubicin and TQR combination studies in the MDA-MB-468_MDR1_ cell line. Timecourses of the mean intracellular concentration of doxorubicin with corresponding standard deviations are shown for each treatment condition in **(A)**. Doxorubicin accumulation increases along with TQR concentrations. Equations (1–3) were fit to the data, and the best-fit models are overlaid on the data (smooth lines) in a. Model parameter fits corresponding to the best-fit models are shown in **(B–D)**. Similar *k*_*EF*_ and *k*_*FB*_ vales are observed across all TQR concentrations. There is a trend of decreasing *k*_*FE*_ values with increasing TQR concentrations **(C)**, consistent with MDR1 inhibition by TQR. Cell counts of MDA-MB-468_MDR1_ following combination treatment with TQR and doxorubicin are show in panels **(E-G)**. In each plot, a fixed concentration of doxorubicin is applied with variable TQR concentrations. These counts are fit with Equations (4–6) as described in section Model Fits, and the best-fit model is overlaid on the cell counts [smooth lines in panels **(E–G)**]. Error bars represent the 95% CI from six experimental replicates for each treatment condition. Model parameters with corresponding 95% CI are shown in **(H–J)** as a function of TQR concentration. For each doxorubicin concentration, the death rate (*k*_*d,a*_ and *k*_*d,b*_) increased with TQR concentration **(H,I)**.

The measured intracellular doxorubicin concentration timecourses for the MDA-MB-468_MDR1_ cell line under doxorubicin monotherapy and combination therapy with TQR are shown in Figure 2A. Intracellular doxorubicin increases with TQR concentration. The average intracellular concentration at the end of each experiment, estimated with the last 10 timepoints, is significantly different among the treatment groups (one-way ANOVA, *p* < 1e-5). Equations (1–3) are fit to these data, and the best-fit models are overlaid on the timecourses. The mean error of the best-fit pharmacokinetic models was 45.6 (±47.4) nM across all timepoints and treatment conditions, and the corresponding model parameters are shown in Figures 2B–D. Increasing TQR concentrations decrease doxorubicin efflux in the MDA-MB-468_MDR1_ cell line in a dose-dependent manner. For example, the efflux rate (*k*_*FE*_) is decreased from 0.216 (±0.028) h^−1^ to 0.046 (±0.008) h^−1^ as TQR increases from 2 to 250 nM (the bounds here and below correspond to the 95% confidence interval of the parameter estimates). *k*_*EF*_ values varied with TQR concentration, all falling within [1.63, 3.46] × 10^−6^ h^−1^.

**Figure 3:**
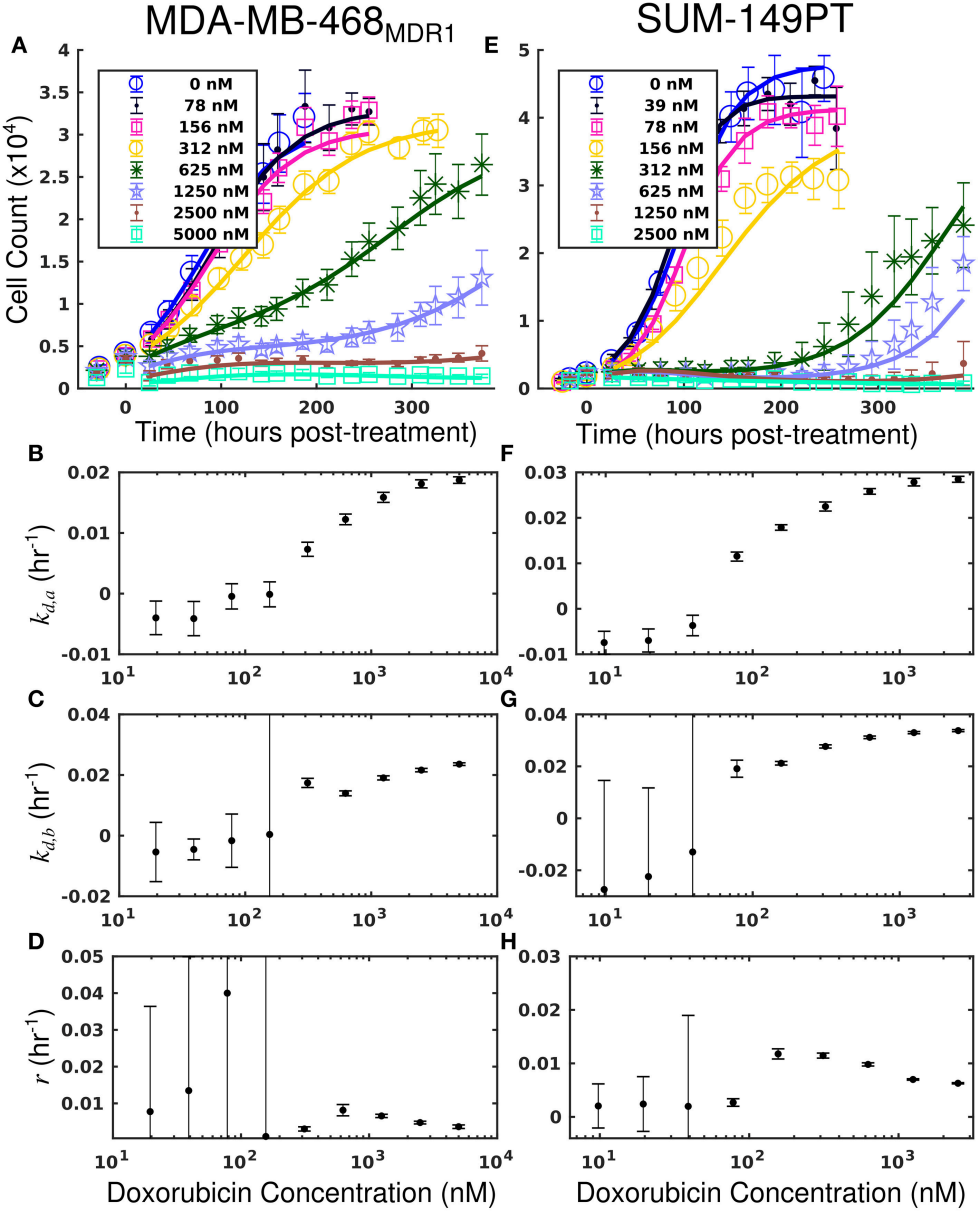
Treatment response in MDA-MB-468_MDR1_ (left column) and SUM-149PT (right column) cell lines under doxorubicin monotherapy. The top row [panels **(A,E)**] shows cell counts over time from treatment response studies for each cell line. For these studies, cells were treated with a fixed concentration of doxorubicin for 24 h. These counts are fit to Equations (4–6) as described in section Model Fits, and the best-fit model is overlaid on the cell counts [smooth lines in **(A,E)**]. Error bars represent the 95% CI from six experimental replicates for each treatment condition. Model parameters with corresponding 95% CI are shown in the bottom three rows as a function of doxorubicin concentration. Panels **(B–D)** show fits from the MDA-MB-468_MDR1_ experiments, and panels **(F–H)** show fits from the SUM-149PT experiments. For each doxorubicin concentration for each cell line, the death rate (*k*_*d,a*_ and *k*_*d,b*_) increased with increasing doxorubicin concentrations.

Treatment response timecourses for the MDA-MB-468_MDR1_ cell line under doxorubicin combination therapy with TQR are shown in Figures 2E–G. Equations (4–6) are fit to these data, and the best-fit models are overlaid on the observed cell counts. Model parameters are shown in Figures 2H–J. For a fixed concentration of doxorubicin, increasing concentrations of TQR incrementally sensitize cells to doxorubicin. For example, at a fixed dose of 156 nM doxorubicin, increasing the TQR concentration from 0 to 250 nM increased the death rate (*k*_*d, a*_) from −0.16 (±0.23) × 10^−2^ h^−1^ to 2.21 (±0.1) × 10^−2^ h^−1^. TQR monotherapy did not affect the growth of these cells as shown in Supplementary Figure 3. Treatment response timecourses of the MDA-MB-468_MDR1_ line to doxorubicin monotherapy are shown in Figure 3A. These data are fit with Equations (4–6), and the best-fit models are overlaid on the observed cell counts. The mean percent error of the best-fit model across all timepoints and treatment conditions is 10.3%. Prior to treatment, the MDA-MB-468_MDR1_ line demonstrated a proliferation rate (*k*_*p*_) of 2.12 (±0.03) × 10^−2^ h^−1^. Treatment response varied smoothly with doxorubicin concentration, and this response is quantified by the parameters in Figures 3B–D. Notably, high variance in parameter estimates is observed as values of *r* approach 0.05 h^−1^ and values of *k*_*d, b*_ approach 0 h^−1^. There exists intrinsic uncertainty at this limit as the rapid dynamics (*r*) coupled with small *k*_*d, b*_ effects cannot be resolved by the current data. This uncertainty in *r* for small *k*_*d, b*_ does not affect model predictions as demonstrated by a sensitivity analysis in previous work (McKenna et al., 2017).

**Figure 4:**
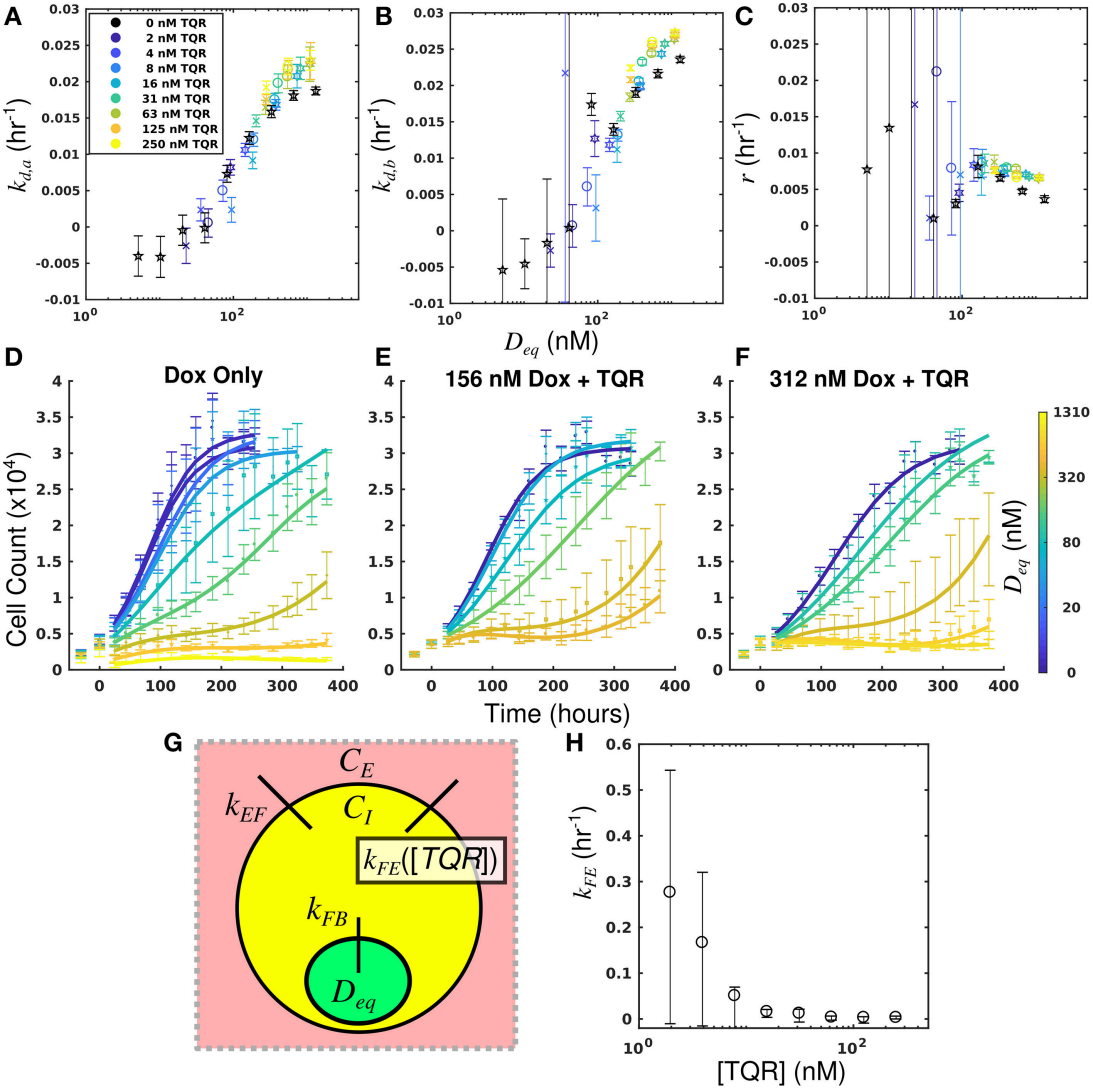
Leveraging equivalent dose to estimate the effect of TQR in the MDA-MB-468_MDR1_ cell line. The equivalent dose for each doxorubicin monotherapy condition was first calculated with the PK model parameters measured in the doxorubicin uptake studies. The equivalent dose statistic was then estimated for each co-treatment condition by matching treatment response parameters from co-treatment conditions to those from doxorubicin monotherapy conditions. Parameter values from all doxorubicin monotherapy and co-treatment conditions are plotted as a function of equivalent dose **(A-C)**. A subset of responses from doxorubicin monotherapy and co-treatment conditions are color-coded to their estimated equivalent dose **(D–F)**. Similar dynamics are observed with similarly-colored data, demonstrating the efficacy of the parameter matching in comparing treatment response timecourses. As TQR impairs the function of the MDR1 pump, we hypothesized the effect of TQR is limited to the *k*_*FE*_ parameter **(G)**. With estimates of equivalent dose for all treatment conditions, the *k*_*FE*_ value for each TQR concentration was estimated with the optimization routine summarized by Equation (7) **(H)**. These values, calculated with treatment response data, agree well with direct measurements of *k*_*FE*_ reported in Figure 2. We note the large confidence intervals are a result of the optimization approach, in which the value (1/*k*_*FE*_) was optimized.

By leveraging the proposed mechanistic model and equivalent dose statistic, *k*_*FE*_ values for each TQR concentration can be estimated using the measured treatment response data and the optimization routine outlined in section Measurement of Pharmacologic Properties With Equivalent Dose. To make these measurements, the equivalent dose for each doxorubicin monotherapy condition was first calculated with the PK model parameters measured in the doxorubicin uptake studies. Specifically, *k*_*FE*_, *k*_*EF*_, and *k*_*FB*_ were measured to be 0.313 h^−1^, 3.08 × 10^−6^ h^−1^ and 0.0212 h^−1^, respectively. The equivalent dose statistic was then estimated for each co-treatment condition. To perform this estimation, treatment response parameters from co-treatment conditions were matched to those from doxorubicin monotherapy conditions (Figures 4A–C). As the equivalent doses for all monotherapy conditions are perfectly known (i.e., *C*_*B*_ = *D*_*eq*_ for doxorubicin monotherapy), the equivalent dose for each co-treatment condition can be estimated with the matching process illustrated in Supplementary Figure 2. Briefly, parameter values are estimated across a range of equivalent doses utilizing parameters from the doxorubicin monotherapy experiments. The equivalent dose for each treatment condition can then be estimated by matching measured parameter values to those estimates. To demonstrate the efficacy of the parameter matching in comparing treatment response timecourses, a subset of responses from doxorubicin monotherapy and co-treatment conditions are color-coded to their estimated equivalent dose (Figures 4D–F). Note similar dynamics for similarly-colored data, indicating the efficacy of the parameter matching in comparing treatment response timecourses. With estimates of equivalent dose for all co-treatment conditions, the *k*_*FE*_ value for each TQR concentration was estimated with the optimization routine summarized by Equation (7). As we hypothesized that the effect of TQR is limited to *k*_*FE*_ (Figure 4G), *k*_*EF*_ and *k*_*FB*_ values were fixed to the values reported above in the optimization routine. The optimized *k*_*FE*_ values for all TQR concentrations are shown in Figure 4H. Decreasing *k*_*FE*_ values were observed with increasing TQR concentrations, matching the measurements from the uptake studies in Figure 2.

The equivalent dose can summarize all treatment conditions in the MDA-MB-468_MDR1_ cell line and is predictive of response. Further, this statistic can be leveraged to quantify the effect of TQR.

### Treatment Response in SUM-149PT Cell Line

**Figure 5:**
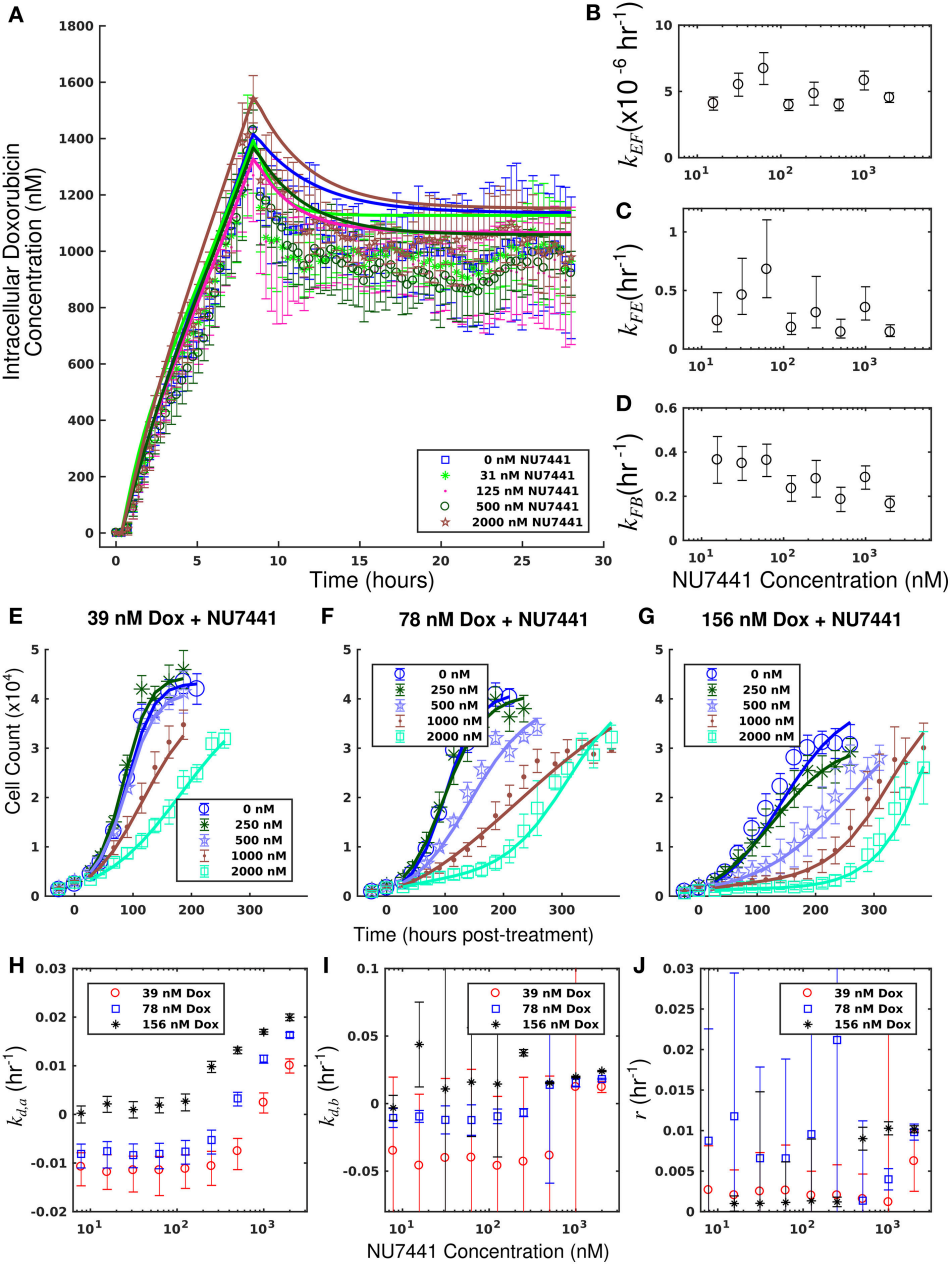
Doxorubicin and NU7441 combination studies in the SUM-149PT cell line. Timecourses of the mean intracellular concentration of doxorubicin with corresponding standard deviations are shown for each treatment condition in a. No significant difference in doxorubicin accumulation was observed as a function of NU7441 concentration. Equations (1–3) were fit to the data, and the best-fit models are overlaid on the data (smooth lines) in **(A)**. Model parameter fits corresponding to the best-fit models are shown in **(B–D)**. For each model parameter, similar vales were observed across all NU7441 concentrations, consistent with the similar intracellular doxorubicin timecourses in **(A)**. Counts of SUM-149PT cells following combination treatment with NU7441 and doxorubicin are show in panels **(E–G)**. In each plot, a fixed concentration of doxorubicin is applied with variable NU7441 concentrations. These counts are fit with Equations (4–6) as described in section Model Fits, and the best-fit models are overlaid on the cell counts [smooth lines in panels **(E–G)**]. Error bars represent the 95% CI from six experimental replicates for each treatment condition. Model parameters with corresponding 95% CI are shown in panels **(H–J)** as a function of NU7441 concentration. For each doxorubicin concentration, the death rate (*k*_*d,a*_) increased with NU7441 concentration **(H)**. The parameters shown in panels **(I,J)** are unable to be resolved with the current data as discussed in section Model Fits.

The measured intracellular doxorubicin concentration timecourses for the SUM-149PT cell line under doxorubicin monotherapy and combination therapy with NU7441 are shown in Figure 5A. NU7441 treatment did not affect intracellular doxorubicin accumulation following treatment. The average intracellular doxorubicin concentration at the end of each experiment, estimated with the last 10 timepoints, did not demonstrate significant differences at the *p* = 0.05 level (one-way ANOVA). Equations (1–3) are fit to the uptake data, and the best-fit model is overlaid on the timecourses. The corresponding model parameters are shown in Figures 5B–D. The mean error of the best-fit pharmacokinetic model was 77.9 (±71.4) nM across all treatment conditions and timepoints. Further, similar values of *k*_*FE*_, *k*_*EF*_, and *k*_*FB*_ are observed across all NU7441 concentrations (Figures 5B–D). Given its effect on DNA-PK, NU7441 is not expected to affect intracellular doxorubicin accumulation.

Treatment response timecourses for the SUM-149PT cell line under doxorubicin co-treatment with NU7441 are shown in Figures 5E–G. Equations (4–6) are fit to these data, and the best-fit models are overlaid on the observed cell counts. Model parameters are shown in Figures 5H–J. For a fixed concentration of doxorubicin, increasing concentrations of NU7441 incrementally sensitized cells to doxorubicin. For example, with a fixed dose of 156 nM doxorubicin, NU7441 concentrations increased the death rate (*k*_*d, a*_) from 0.25 (±0.16) × 10^−2^ h^−1^ to 2.00 (±0.06) × 10^−2^ h^−1^. NU7441 monotherapy did not affect the growth of these cells as shown in Supplementary Figure 3. Treatment response timecourses of the SUM-149PT line to doxorubicin monotherapy are shown in Figure 3E. These data are fit with Equations (4–6), and the best-fit models are overlaid on the observed cell counts. The mean percent error of the best-fit model across all treatment conditions is 11.9%. Prior to treatment, the SUM-149PT line demonstrated a proliferation rate (*k*_*p*_) of 2.58 (±0.03) × 10^−2^ h^−1^. Treatment response varied smoothly with doxorubicin concentration, and this response is quantified by the parameters in Figures 3F–H.

**Figure 6:**
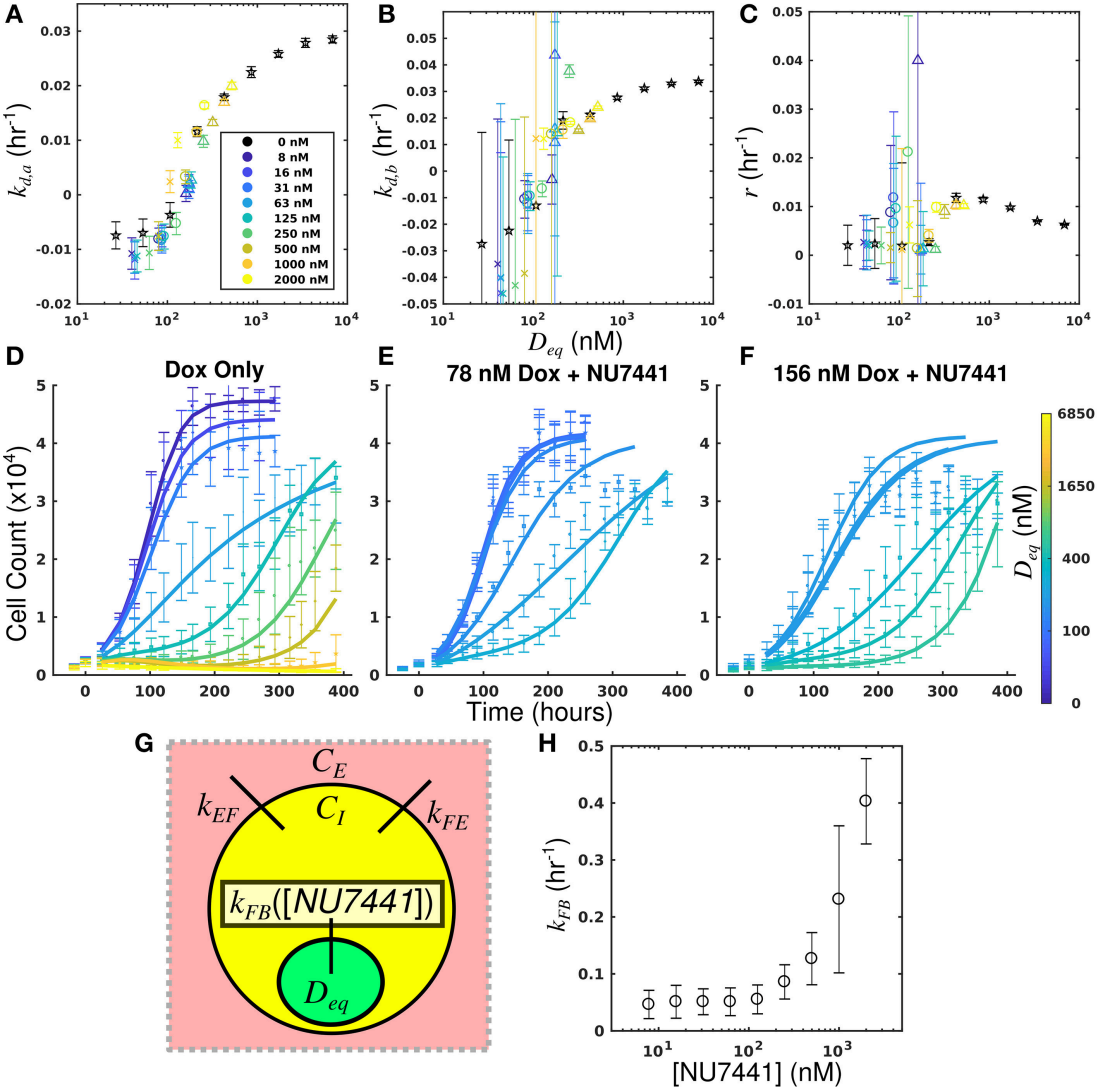
Leveraging equivalent dose to estimate the effect of NU7441 in the SUM-149PT cell line. The equivalent dose for each doxorubicin monotherapy condition was first calculated with the PK model parameters measured in the doxorubicin uptake studies. The equivalent dose statistic was then estimated for each co-treatment condition by matching treatment response parameters from co-treatment conditions to those from doxorubicin monotherapy conditions. Parameter values from all doxorubicin monotherapy and co-treatment conditions are plotted as a function of equivalent dose **(A-C)**. A subset of responses from doxorubicin monotherapy and co-treatment conditions are color-coded to their estimated equivalent dose **(D–F)**. Similar dynamics are observed with for similarly-colored data, demonstrating the efficacy of the parameter matching in comparing treatment response. As NU7441 impairs the function DNA-PK, we hypothesized the effect of NU7441 is limited to the *k*_*FB*_ parameter **(G)**. With estimates of equivalent dose for all treatment conditions, the *k*_*FB*_ value for each NU7441 concentration was estimated with the optimization routine summarized by Equation (7). Increasing values of *k*_*FB*_ are observed with increasing NU7441 concentrations, indicating an increase in functional drug bound **(H)**. These values cannot be directly observed with the uptake study, demonstrating the utility of the equivalent dose in estimating parameters that cannot be directly measured with current techniques.

By leveraging the proposed mechanistic model and equivalent dose statistic, *k*_*FB*_ values for each NU7441 concentration can be estimated using the measured treatment response data and the optimization routine summarized by Equation (7). To make these measurements, the equivalent dose for each doxorubicin monotherapy condition was first calculated with the PK model parameters measured in the doxorubicin uptake studies. Specifically, *k*_*EF*_, *k*_*FE*_, and *k*_*FB*_ were measured to be 4.00 × 10^−6^ h^−1^ and 0.165 h^−1^, and 0.236 h^−1^, respectively. These were calculated by fitting the SUM-149PT uptake studies assuming constant parameters for all NU7441 concentrations. The equivalent dose statistic was then estimated for each co-treatment condition. To perform this estimation, treatment response parameters from co-treatment conditions were matched to those from doxorubicin monotherapy conditions (Figures 6A–C). As the equivalent doses for all monotherapy conditions are perfectly known, the equivalent dose for each co-treatment condition can be estimated with this matching process. To demonstrate the efficacy of the parameter matching in comparing treatment response timecourses, a subset of responses from doxorubicin monotherapy and co-treatment conditions are color-coded to their estimated equivalent dose (Figures 6D–F). Note similar dynamics for similarly-colored data. With estimates of equivalent dose for all co-treatment conditions, the *k*_*FB*_ value for each NU7441 concentration was estimated with the optimization routine summarized by Equation (7). As we hypothesized that the effect of NU7441 is limited to *k*_*FB*_(Figure 6G), *k*_*FE*_ and *k*_*EF*_ values were fixed to the values reported above in the optimization routine. The optimized *k*_*FB*_ values for all NU7441 concentrations are shown in Figure 6H. Increasing *k*_*FB*_ values were observed with increasing NU7441 concentrations, indicating the functional increase in drug with NU7441, mediated through its effect on DNA-PK. We note that the DNA repair pathway affected by NU7441 is not directly measured in the uptake studies. Recall from section Equivalent Dose that *k*_*FB*_ is a mixed measure of doxorubicin binding and DNA repair and describes the functional net binding rate. Thus, these values cannot be directly compared to the values extracted from the uptake study.

The equivalent dose can summarize all treatment conditions in the SUM-149PT cell line and is predictive of response. Further, this statistic can be leveraged to quantify the specific effect of NU7441 with observed treatment response data.

### Comparison of MDA-MB-468_MDR1_ and MDA-MB-468_H2B_

**Figure 7:**
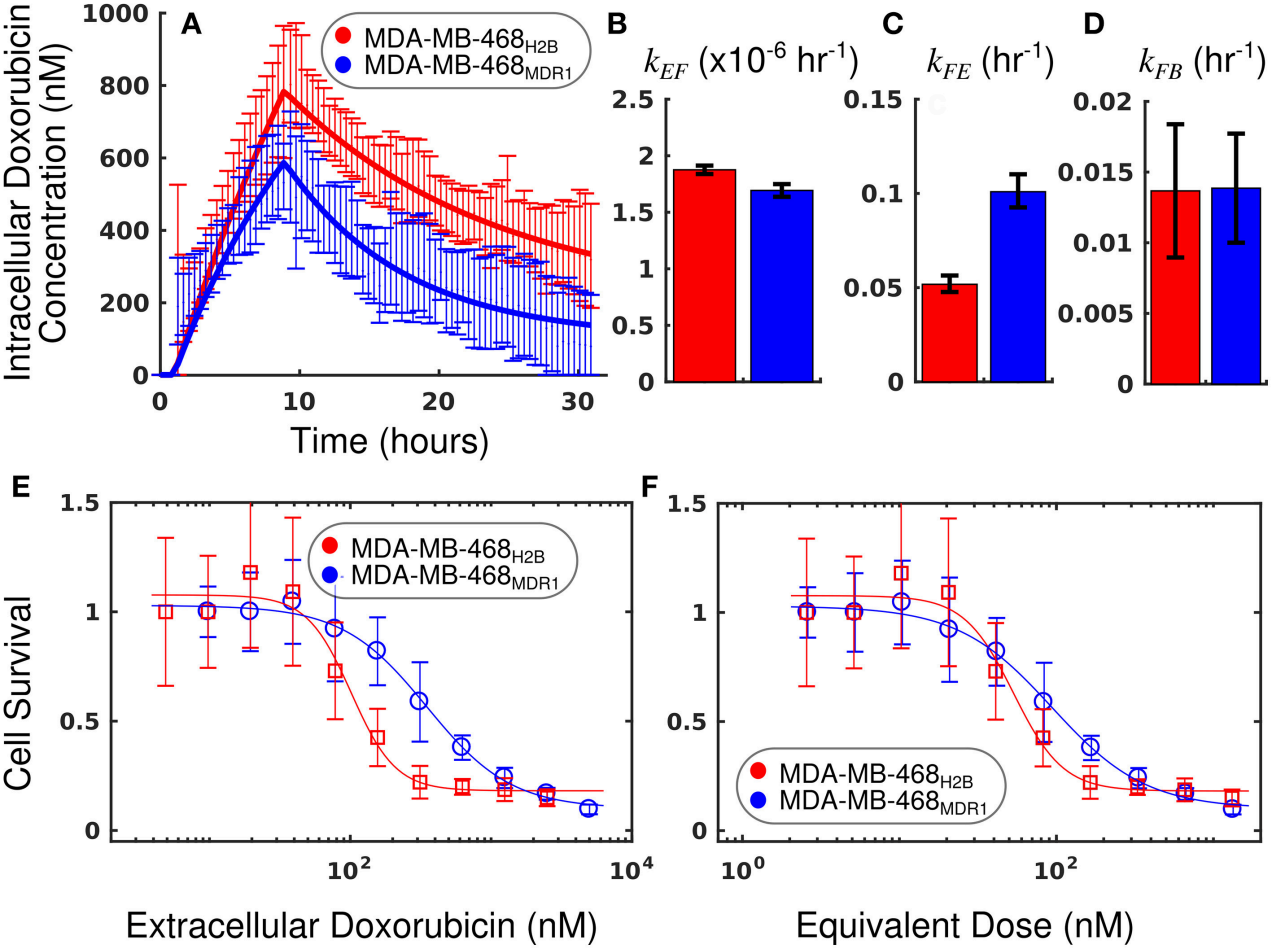
Comparison of MDA-MB-468_H2B_ and MDA-MB-468_MDR1_ cell lines using equivalent dose. The intracellular doxorubicin concentration with 95% CI for each cell line is shown in **(A)**. The MDA-MB-468_H2B_ line demonstrates increased intracellular accumulation of doxorubicin relative to the MDA-MB-468_MDR1_ line. Equations (1–3) are fit to the doxorubicin uptake data, and the best-fit models are overlaid on the data in a (smooth line). The corresponding parameters with 95% CI are shown in **(B–D)**. The MDA-MB-468_H2B_ data are shown in red, and the MDA-MB-468_MDR1_ data are shown in blue. Notably, the efflux of drug from the MDA-MB-468_MDR1_ (*k*_*FE*_) line is significantly greater than the corresponding rate in the MDA-MB-468_H2B_ line (*p* < 0.05). Treatment response is traditionally summarized by cell survival and plotted against applied drug concentration. The cell count relative to control for each cell line is shown as a function of extracellular doxorubicin concentration and equivalent dose in **(E,F)**, respectively. While a significant difference is observed when comparing these cell lines *via EC*_50_calculated with the extracellular doxorubicin concentration, no significant difference is observed when comparing the *EC*_50_ statistic derived from the equivalent dose. The equivalent dose can account for the differing pharmacokinetic properties to reveal similar doxorubicin pharmacodynamics in these cell lines.

The measured intracellular doxorubicin concentration timecourses with accompanying best-fit models for the MDA-MB-468_H2B_ and MDA-MB-468_MDR1_ cell lines are shown in Figure 7. Decreased doxorubicin accumulation was observed in the MDA-MB-468_MDR1_ cell line relative to its parental line, MDA-MB-468_H2B_. Notably, drug efflux was significantly elevated in the MDA-MB-468_MDR1_ line relative to its parental line with *k*_*FE*_ values of 1.01 (±0.08) × 10^−1^ h^−1^ and 0.52 (±0.04) × 10^−1^ h^−1^, respectively (*p* < 0.05). The mean errors of the best-fit pharmacokinetic models across all timepoints were 44.7 and 58.7 nM for the MDA-MB-468_H2B_ and the MDA-MD-468_MDR1_ lines, respectively.

The survival of each cell line 72 h following treatment is compared as a function of extracellular doxorubicin concentration and equivalent dose in Figures 7E,F. The *EC*_50_ as measured with the extracellular doxorubicin concentration for the MDA-MB-468_H2B_ and the MDA-MB-468_MDR1_ are 101.6 (±28.9) and 350.6 (±109) nM, respectively. These measures indicate that there is a statistically significant difference between these cell lines (*p* < 0.05, *t*-test). The *EC*_50_ as measured with the equivalent dose for the MDA-MB-468_H2B_ and the MDA-MB-468_MDR1_ lines are 53.3 (±15.1) and 93.7 (±29.2) nM, respectively. These values are not different at *p* = 0.05 (*t*-test), indicating the similarity of these lines. Indeed, the only difference between cell lines is the overexpression of the MDR1 efflux pump. The intrinsic sensitivity of these cell lines to treatment should remain similar, and the equivalent dose reflects this similarity. The response of the MDA-MB-468_H2B_ and MDA-MB-468_MDR1_ cell lines are not significantly different as measured by *D*_*eq*_.

## Discussion

We have proposed and demonstrated the utility of a mathematical modeling framework to quantify pharmacologic properties. We further proposed a new metric, the equivalent dose (*D*_*eq*_), which provides a biochemically-based measure of treatment effect. With the data presented here, we show that a mechanistic mathematical model of treatment response can succinctly summarize a range of treatments to allow for more precise comparison of treatment response among cell lines. Further, we have shown how this model provides quantitative biological insight into the biochemical drivers of treatment response. We demonstrate that a mathematical modeling framework allows for quantification of pharmacologic processes through population-scale measurements.

Treatment response is driven by cell-line specific pharmacologic properties. Conventional summary statistics of treatment response data often conflate these pharmacologic properties, limiting their utility. To more effectively advance the study of treatment response, methods that explicitly consider this variability are needed to more precisely quantify biological drivers of treatment response. While previous treatment response assays provide insight in the relative sensitivity of a cell line to therapy (Fallahi-Sichani et al., 2013), the proposed approach quantifies specific drivers of treatment sensitivity. Through the approach proposed in this work, we demonstrate how intracellular pharmacologic properties can be quantified using limited data from population-level observations of treatment response.

This work is limited by its use of doxorubicin, which is intrinsically fluorescent, thereby allowing for the uptake model to be fit with experimental data. However, this approach need not be limited to fluorescent drugs. With appropriate experimental design, the approach summarized by Equation (7) can be leveraged to quantify any of the rates proposed in the model. Indeed, the optimized values of doxorubicin efflux in the MDA-MB-468_MDR1_ line in Figure 4 are similar to those values measured by the uptake assay in Figure 2. Further, the effect of NU7441 in altering pharmacokinetics was quantified using only the treatment response data, as this effect cannot be directly measured in the uptake assay. Importantly, this work demonstrates that all treatment conditions collapse onto a single, smooth trajectory through parameter space as a function of equivalent dose, and this property can be leveraged to provide quantitative insight into the biological drivers of treatment response. While cell lines could not be compared without precise estimates of all model parameters, this approach can nevertheless be used to quantify therapeutic perturbations within a given cell line. It is straightforward to extend the proposed modeling approach as a means to more precisely quantify the effects of other parameters in the experimental microenvironment (e.g., how does pH or a specific nutrient concentration affect treatment response?). In this way, these variables can be mapped onto a unified treatment response framework to more efficiently advance precision medicine approaches. More generally, the approach outlined in this work demonstrates how mathematical modeling can be used as a “filter” to derive more specific measures from experimental data to advance systems biology.

Therapies that target PK/PD pathways offer the potential to sensitize cells to cytotoxic therapies, increasing the efficacy of therapy and allowing for lower doses of such therapeutics. The approach proposed in this work provides a means to quantify the respective contributions of PK/PD pathways, providing mechanistic insight into treatment response. This approach differs from current methods used to assess drug synergism and antagonism (Chou, 2006; Jones et al., 2014; Foucquier and Guedj, 2015; Chen and Lahav, 2016; Lederer et al., 2018). These methods have great utility in discovering and quantifying drug interactions; however, they cannot be leveraged to understand the mechanisms underlying the identified synergy/antagonism. While other methods have leveraged mechanistic data to identify synergy (Al-Lazikani et al., 2012; Gao et al., 2017; Yin et al., 2018), the proposed equivalent dose framework provides quantitative mechanistic insight into intracellular drug effects and allows for predictions of treatment response under a variety of treatment conditions. We posit that this mechanistic approach could facilitate clinical translation of combination therapies. Notably, therapeutic approaches intended to sensitize tumors to doxorubicin have demonstrated great preclinical activity; however, their efficacy has been limited in clinical trials. Specifically, negative results have been seen with TQR due to excess toxicities and inactivity (Pusztai et al., 2005; Fox and Bates, 2007). Similarly, DNA-PK inhibitors such as NU7441 have yet to demonstrate an effect clinically despite their preclinical promise (Zhao et al., 2006; Helleday et al., 2008; Davidson et al., 2013). We posit that the proposed modeling framework can be used to identify more effective strategies for dosing and assessing these therapeutics. In particular, the proposed modeling approach can provide *precise* guidance on the necessary dose adjustments to achieve a desired effect in the context of combination therapy. As we have demonstrated, a target equivalent dose can be achieved in a variety of ways. For example, the extracellular drug concentration timecourse can be tuned to reach a specified equivalent dose. Alternatively, the same equivalent dose can be achieved by altering cell line pharmacologic properties through sensitizers with concomitant changes in the extracellular doxorubicin timecourse. While realizing this goal *in vivo* will require a more complete model of treatment response (i.e., one that incorporates plasma pharmacokinetics and organ system toxicities), we have demonstrated the proposed model to be robust to various doxorubicin treatments and is general to sensitizing agents.

While the results of this study are promising, several limitations exist in the current approach. The first order pharmacokinetics model assumes static kinetic rates throughout the experiment, and the pharmacokinetic rates were investigated at only a single concentration. These rates are calculated as an average over all observed cells, not accounting for intercellular heterogeneity. Further, these kinetics may saturate as a function of doxorubicin concentration. This method remains to be validated in additional cell lines with other pharmacologic targets to address its generalizability. Additional properties of *in vitro* assays not explicitly considered in the current model have been shown to confound observed effects. For example, local cell densities have been found to affect treatment response (Greene et al., 2016). Finally, this model is deterministic and does not consider either population heterogeneity or cell evolution. Despite these limiting assumptions, we note the accuracy of the equivalent dose in summarizing population-level response to a range of doxorubicin treatment conditions.

In this work, we have demonstrated how mathematical modeling can be leveraged to quantify PK/PD pathways and more precisely compare treatment response among cell lines. It is the ultimate goal of precision cancer therapy to deliver the optimal therapy on the optimal schedule for the individual patient (McKenna et al., 2018). A necessary step toward this goal is to establish a robust functional relationship between applied treatment and subsequent response. The present study demonstrates the utility of the modeling framework and provides additional evidence that the response to therapy is *predictable*. In summary, analysis of treatment response data with mechanistic models can effectively quantify the effects of various biological and pharmaceutical perturbations on treatment response.

## Author Contributions

MM and TY conceived the experiments. MM conducted the experiments under the guidance of TY. MM analyzed all results. JW aided MM in developing the numerical methods to fit the proposed models. All authors reviewed the manuscript.

### Conflict of Interest Statement

The authors declare that the research was conducted in the absence of any commercial or financial relationships that could be construed as a potential conflict of interest.
