# Supplementary material for: Leveraging Mathematical Modeling to Quantify Pharmacokinetic and Pharmacodynamic Pathways: Equivalent Dose Metric
Source: Front Physiol. 2019 May 22;10:616. doi: 10.3389/fphys.2019.00616 (PMC6538812; doi:10.3389/fphys.2019.00616)
Supplement: Supplementary file 1 [file Image_1.pdf]

Supplementary Material

1 Supplementary Figures

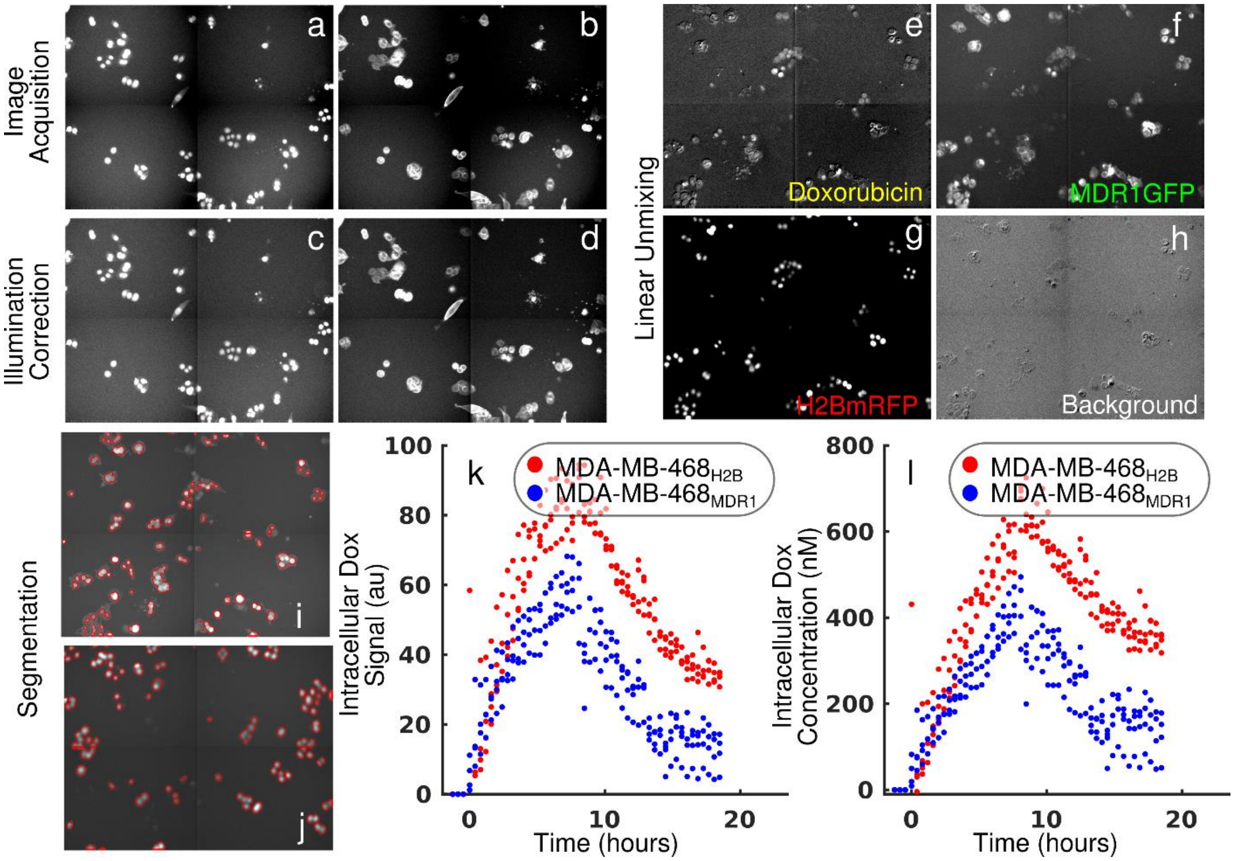

Supplementary Figure 1. Doxorubicin image processing pipeline. Five images ($I_1 \ldots I_5$) of each well at each timepoint are collected. Sample images of I_4 and I_5 at a representative timepoint are shown in a and b, respectively. The uneven illumination of these images is corrected through use of the image illumination function described in section Doxorubicin Uptake Imaging and Image Processing. Corrected images of a and b are shown in c and d, respectively. A linear unmixing approach is employed to estimate the signal from each fluorophore present in the experimental system. Sample reconstructed signals (i.e., S_{Dox}, S_{MDR}, S_{H2B}, and $S_{background}$) are shown in e-h. Cells are segmented *via* a threshold approach (red outline in i and j). The doxorubicin signal in the intracellular space over time can then be extracted from these images (k) and converted into concentration timecourses (l) to be fit by Eqs. (1) – (3).

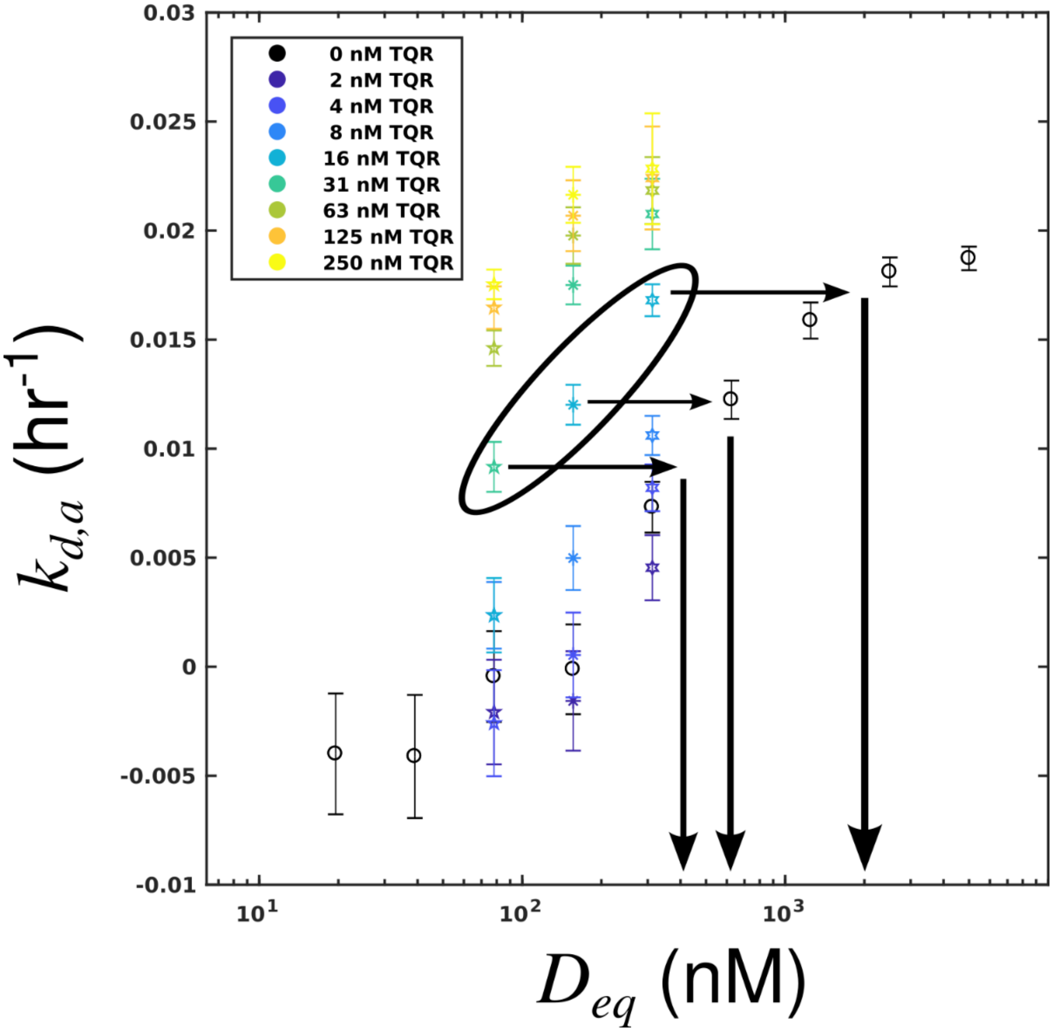

Supplementary Figure 2. Calculating D_{est}. The treatment response model (i.e., Eqs. (4) – (6)) is fit to all data (doxorubicin monotherapy and co-treatment with a sensitizer), yielding a set of parameters, $p = [k_{d,a}, k_{d,b}, r]$, for each treatment condition. These parameters are plotted with respect to equivalent dose (D_{eq}), as calculated with the k_{EF}, k_{FE}, and k_{FB} parameters derived from the monotherapy uptake studies. The black points are parameter fits to the doxorubicin monotherapy conditions. The colored data represent parameter fits from three experiments each with a unique, fixed doxorubicin concentration and variable sensitizer concentration. The equivalent dose for each combination condition is then estimated as follows. By definition, we assume that each unique treatment response timecourse corresponds to a specific equivalent dose, and response varies smoothly with equivalent dose. Thus, all treatment response data, as quantified by the parameters *p*, should fall along a smooth continuum. The parameter fits from co-treatment conditions are mapped to the doxorubicin-only treatment (right-facing arrows). The equivalent dose (D_{est}) for each sensitizer condition is the point of intersection between the combination therapy and monotherapy data (down-facing arrows). With estimates of the equivalent dose for each co-treatment condition, Eq. (7) can be leveraged to optimize any parameter under investigation (i.e., k_{EF} and k_{FB} for each TQR and NU7441 concentration, respectively). Parameter values between data points from doxorubicin monotherapy experiments are estimated *via* a local linear interpolation.

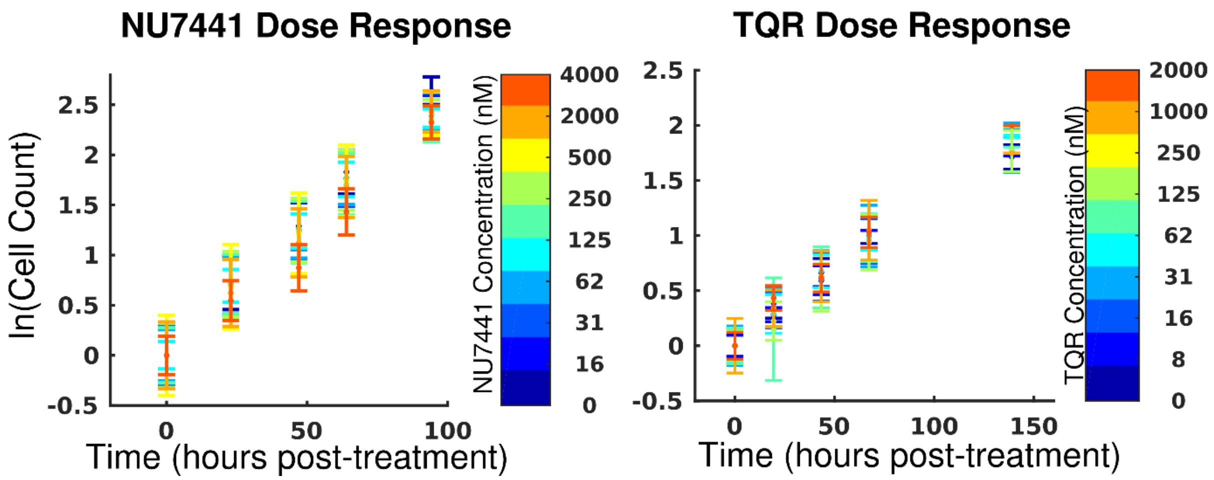

Supplementary Figure 3. TQR-only and NU7441-only control data for the MDA-MB-468$_{\mathbf{MDR1}}$ and SUM-149PT cell lines, respectively. Cell counts with corresponding standard deviations over time under treatment with a range of TQR and NU7441 concentrations are shown. These data demonstrate no significant difference in cell counts under monotherapy with either TQR or NU7441. The effect of these therapies is limited to co-treatment with doxorubicin.
